# Weight Watching and the Effect of Landscape on Honeybee Colony Productivity: Investigating the Value of Colony Weight Monitoring for the Beekeeping Industry

**DOI:** 10.1371/journal.pone.0132473

**Published:** 2015-07-06

**Authors:** Antoine Lecocq, Per Kryger, Flemming Vejsnæs, Annette Bruun Jensen

**Affiliations:** 1 University of Copenhagen, Department of Plants and Environmental Sciences—PLEN, 1871 Frederiksberg C, Denmark; 2 Aarhus University, Department of Agroecology—Entomology and Plant Pathology, 4200 Slagelse, Denmark; 3 Danish Beekeepers Association, Fulbyvej 15, 4180 Sorø, Denmark; Ghent University, BELGIUM

## Abstract

Over the last few decades, a gradual departure away from traditional agricultural practices has resulted in alterations to the composition of the countryside and landscapes across Europe. In the face of such changes, monitoring the development and productivity of honey bee colonies from different sites can give valuable insight on the influence of landscape on their productivity and might point towards future directions for modernized beekeeping practices. Using data on honeybee colony weights provided by electronic scales spread across Denmark, we investigated the effect of the immediate landscape on colony productivity. In order to extract meaningful information, data manipulation was necessary prior to analysis as a result of different management regimes or scales malfunction. Once this was carried out, we were able to show that colonies situated in landscapes composed of more than 50% urban areas were significantly more productive than colonies situated in those with more than 50% agricultural areas or those in mixed areas. As well as exploring some of the potential reasons for the observed differences, we discuss the value of weight monitoring of colonies on a large scale.

## Introduction

Humans have lived alongside honey bees for thousands of years. Apiculture, the maintenance of honey bee colonies by humans, has faced a number of challenges in recent years. While our dependence on honey bees has vastly increased, 80% of global agricultural pollination services can be attributed to the European Honey Bee, *Apis mellifera* [1] and 52 of the 115 leading global food commodities depend on honey bee pollination for either fruit or seed set [2], managed colonies in both Europe and North America especially have declined [2]. In the US, the number of honey bee colonies dropped 61% between 1947 and 2008 [2]. In Europe, colony numbers decreased from over 21 million in 1970 to about 15.5 million in 2007 [3]. Our accuracy in estimating the value of pollination services at the national or global scale is still a subject of some debate [4]. However, there are no doubts that currently, the demand for pollination services is overshadowing any potential growth of global honeybee stocks [5, 6].

Various pests, diseases, parasites, predators and their interactions can all adversely affect managed honey bee productivity and survival [2, 7–9]. Other factors, most likely of human origin, influencing honey bee populations include pesticides, weather and climate, and international trade [7, 8, 10]. Laboratory and field based research into the nutritional requirements of honey bees [11, 12], their foraging habits as well as landscape spatial use [13–15], have shed light as to some of the effects that our changing agricultural practices can have on the health and abundance of honey bees. For example, recent studies found that resistance to a parasite increased with increased protein levels in pollen. While polyfloral blends are not necessarily better than the most protein-rich monofloral pollen, providing different floral resources could counteract the effect of low protein pollen on infected worker survival [11]. Furthermore the quality of pollen fed to honey bees infected with *Nosema ceranae* could affect the spore production of the parasite [16].

With the growth of urbanisation, more studies have focused their efforts towards the effects of the urban environment on pollinator sensitivity, abundance and diversity [15, 17–20] with variable results. Banaszak-Cibicka and Zmihorski [21] concluded that the city could be an important habitat for bee species and that species diversity and richness remained stable across the urban gradient of their study site. Similarly, Lowenstein et al. [22] found that both bee abundance and richness increased in neighbourhoods with higher human population density. On the other hand there also seems to be some evidence that bee species richness and/or abundance could be negatively affected by urbanization [18, 19, 23, 24]. While these studies tend to focus on wild, native and/or solitary pollinators, the development of urban apiculture has engendered more questions as to the value and benefits of keeping honey bees in cities and the dangers of agricultural intensification to pollinator health.

The monitoring of honeybee colonies over long periods of time can result in potential long-term trend forming data, which could be regularly analysed and used for the formulation of new hypotheses or predictions [14, 25, 26]. Recent advances in precision and automated technologies mean that scientists now have the opportunity to refine old models and extract more and more meaningful data from colonies [27, 28]. The weighing of colonies, as an indication of health and productivity, has been suggested since the 1950s [29]. Previous research has shown that honey and pollen quantities, notably honey production, are the factors most correlated with colony weight [30]. For a number of years, automated scales distributed to apiaries around Denmark, have been collecting data on the weight of colonies under normal management regimes and on their local environmental conditions. The aim of this study was to determine what valuable information could be drawn out from scale data at the national level. Coupled with environmental variables and land type data, we investigated the effect of the immediate landscape on colony productivity.

## Materials and Methods

### The scales and data collection

**Fig 1:**
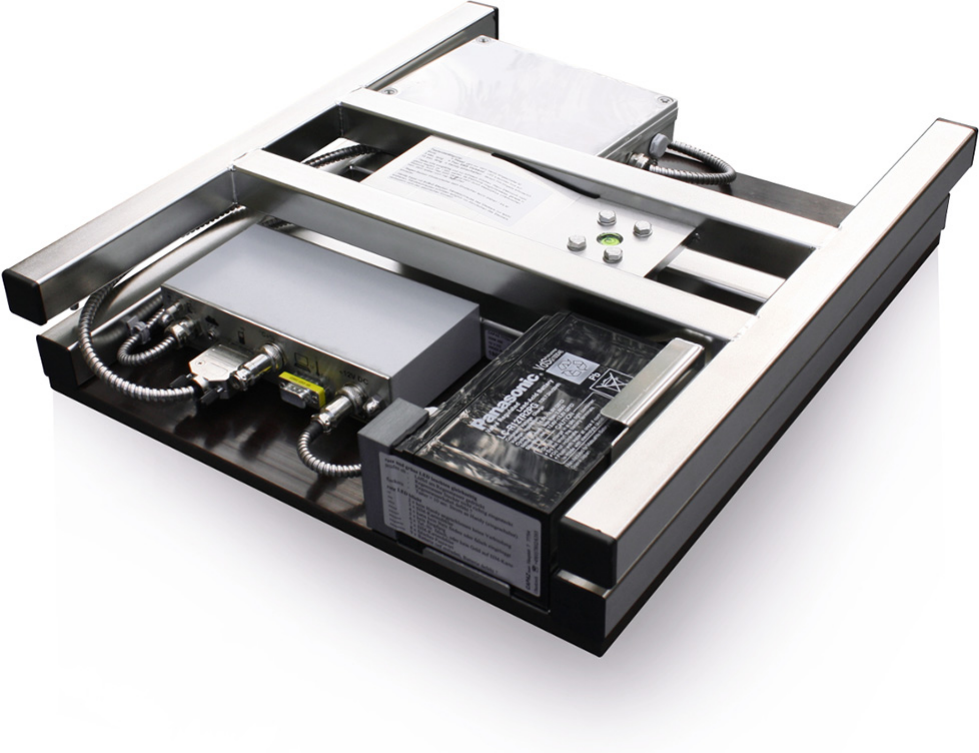
The Capaz scale before installation. The Capaz hivescale is an H-shaped platform made from aluminium and stainless steel, with the dimensions 420 x 480 x 86 mm (long x wide x high). Data are transmitted by cell phone. The rechargeable battery (12 V) lasts for 200 days. Ambient temperature and humidity are measured by default. Additional equipment is the rain collector and brood temperature sensor. Changes of the standard setup of the scale are done via the computer software. Photo: Capaz.

The Capaz hive scale is a 420 x 480 x 86 mm platform made of aluminium and stainless steel (Fig 1). The scale can weigh up to 200 kg with a precision of 100 grams. Weight (mass), ambient temperature, humidity are measured by default. At the end of each day the data from the Capaz scales were sent by a cell phone, as an SMS which was then converted to an email and read by software, which stores and manages the data [27]. Weight records were collected at two hours intervals, with the first record at 6:00 and the last one at 22:00 for each day. All the data used in this study, and further records are available directly from www.stadevægt.dk and www.mybees.buzz and exportable to Excel.

### The colonies

**Fig 2:**
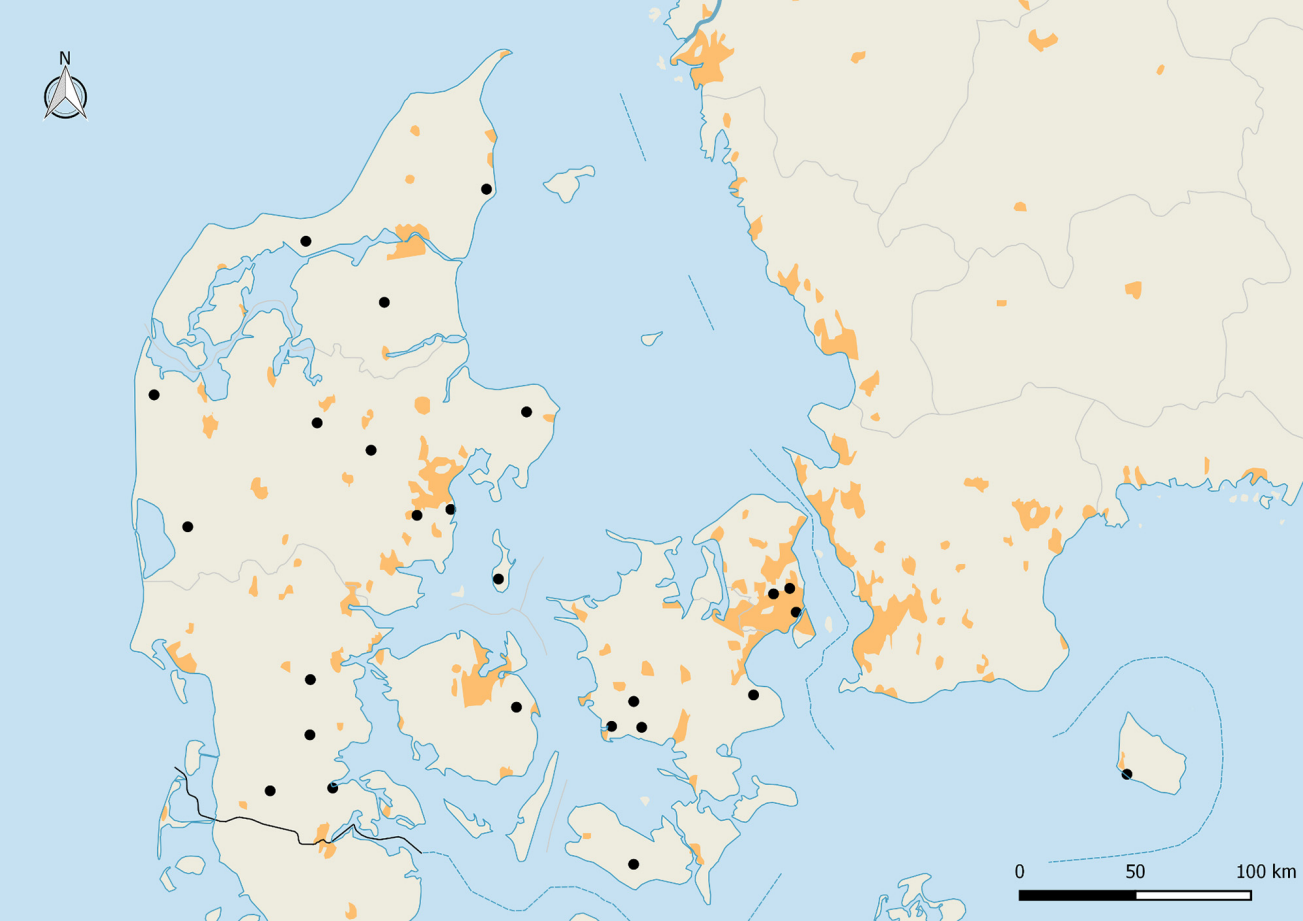
Map of Denmark with the location of the weight scales distributed throughout the country. Darker patches are an indication of major urban areas. Derived from 2002–2003 MODIS satellite data at 1 km resolution [43].

Thirty one colonies, distributed around Denmark, were fitted with a hive scale, featured in the database and had their location mapped. Thirteen of the thirty-one scales fitted to the colonies were part of a monitoring programme set up by the Danish Beekeepers Association [DBA]. The rest of the scales were privately owned. Ultimately, only 26 of the colonies (Fig 2) were selected after data manipulation as described below.

### Data manipulation

In order to deal with the raw data provided by the hive scales, some modifications were essential. The central purpose was to remove any management effect on the colony weights, such as expansion with empty supers, addition of food or removal of honey. In effect, as close as possible, the data represent the collected nectar and pollen, as it is transformed into biomass for each colony in each year. To achieve this in a reproducible way, we systematically removed any weight change exceeding +/-3kg between each two bi-hourly record. The +/-3 kg change indicating more than what bees can achieve in this period, and therefore pointing to manipulations of the colony by the beekeeper. For each year, we recorded a starting point, with whatever the original weight of each colony was on the New Year at 6:00. The bi-hourly records of weights and changes were subsequently averaged for each day to consolidate the data. After analysing the data graphically, any abnormally behaved colonies and / or years (non-existent or negative weights for extended periods of time) were removed to avoid compromising trends and tendencies. Finally, some of the colonies were moved to heather areas in the months of August and September of some years. These data were removed to avoid misinterpretation of seasonal or geographical variation.

This resulted in a total of 71 records from the colonies; 14 in 2010, 16 in 2011, 17 in 2012 and 24 in 2013.

### Environmental variables

Each scale was provisioned with thermometers and precipitation meters, but the data was too variable to be of use, mainly due to inconsistent placement of the equipment and frequent failures. Therefore, rainfall and temperature averages procured from the Danish meteorological institute (DMI at dmi.dk) were used. Monthly averages for the whole of Denmark were provided in the archives for all years investigated. National averages were opted for, since the seasonal variables used in this study (rainfall and temperature) did not vary significantly over the study area.

### Location mapping

All locations were layered on top of the CORINE 2006 land data base using GRASS GIS 7.0.0. The European Environmental Agency (EEA) provides the CORINE (COoRdinate INformation on the Environment) land data base, a pan-European land cover/ land use map for non-commercial use. The resolution of the data is 100 x 100 metres across Denmark. Landscape structure was assessed at 1km and 3km radii surrounding each colony. For habitat typology, the CORINE data contains different levels of resolution. Level 1 resolution uses the broad definitions of agricultural areas, artificial areas, forest and semi natural areas, water bodies and wetlands to categorize the landscape. Due to the general lack of colonies located in natural areas or wetlands, we only analysed the effect of the proportions of agricultural areas and artificial areas on the weight of the colonies, and we also subsequently grouped the Level 1 classes into the following categories:

**Agricultural** = More than 50% of the surrounding designated landscape composed of agricultural areas

**Urban** = More than 50% of the surrounding designated landscape composed of urban areas

**Mixed** = A combination of landscape types surrounding the apiary with no single habitat type representing more than 50% of the total landscape.

The aforementioned 71 annual recordings were thus broken down into 49 in agricultural landscapes, 12 in urban landscapes and 10 in mixed landscapes at a radius of 3km around the apiary, and 47 in agricultural landscapes, 14 in urban landscapes and 10 in mixed landscapes at a radius of 1km around the apiary

### Statistical analysis

The productivity of the honey bee colonies was compared using a One-Way ANOVA (Analysis Of Variance) procedure, and pairwise comparisons of the years were done with a Tukey Pairwise Comparison test on the average annual weights of the colonies. To estimate the link between environmental variables and colony weights, a Pearson’s correlation coefficient was estimated, and the data was grouped seasonally to obtain season weight averages. **Spring** = April, May, June; **Summer** = July, August, September; **Autumn** = October, November, December; **Winter** = January, February, March.

The effect of landscape on the weight of the colonies was tested with a Linear Mixed Model with pairwise comparisons of the years and landscape types. We analysed the effect of the proportions of agricultural areas, artificial areas, and our grouped categories. We used Landscape (proportions or categories), Month, Year and Landscape x Month as fixed factors with Colony ID and Colony ID x Year as random factors. All statistical analyses were performed in SPSS Statistics 22 (SPSS Inc.; Chicago, USA) and Minitab version 17.

## Results

### Overview of the scales data

**Fig 3:**
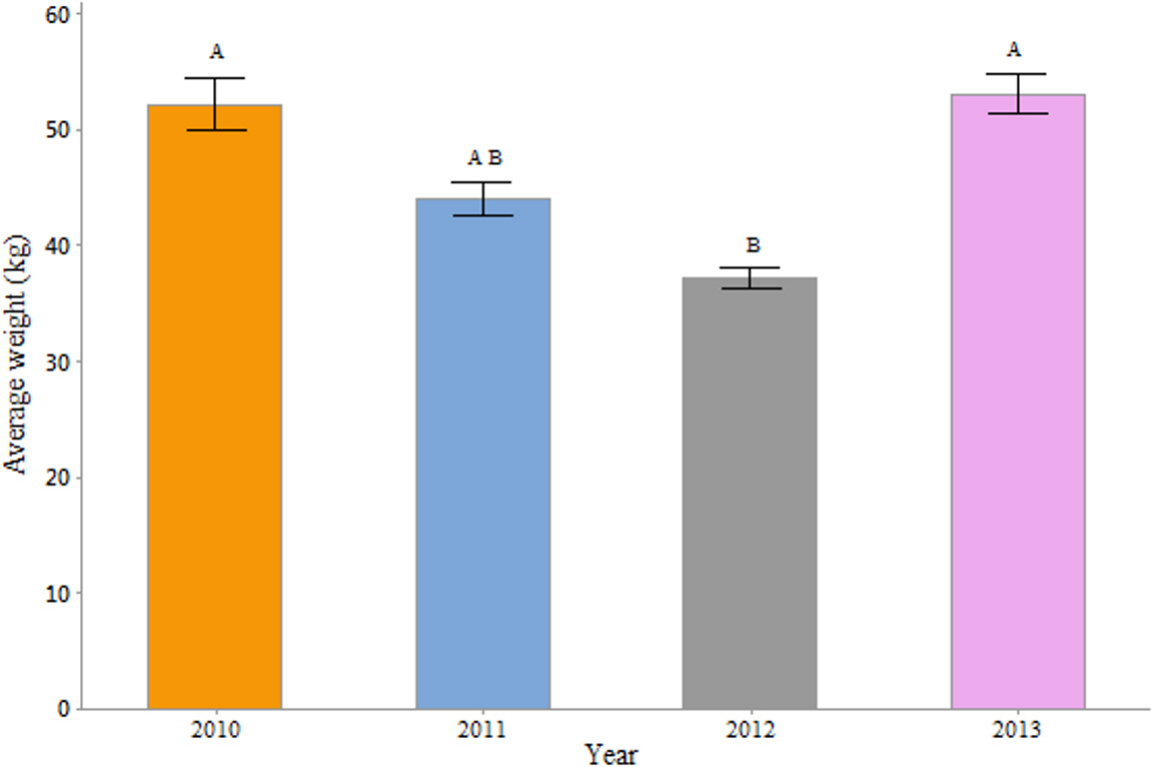
Average weight in kilogram by year for all hives in the Danish database. Means that do not share a letter are significantly different.

The weights of all the hives around Denmark were averaged year by year to estimate basic colony productivity on a national scale. There was a significant difference in weight between the years (One-Way ANOVA; F = 5.63, P = 0.002) (Fig 3). In the year 2012 (n = 17; mean = 37.46) colonies were significantly less productive compared to 2010 (n = 14; mean = 52.06; P = 0.013), and 2013 (n = 24; mean = 52.41; P = 0.003).

**Fig 4:**
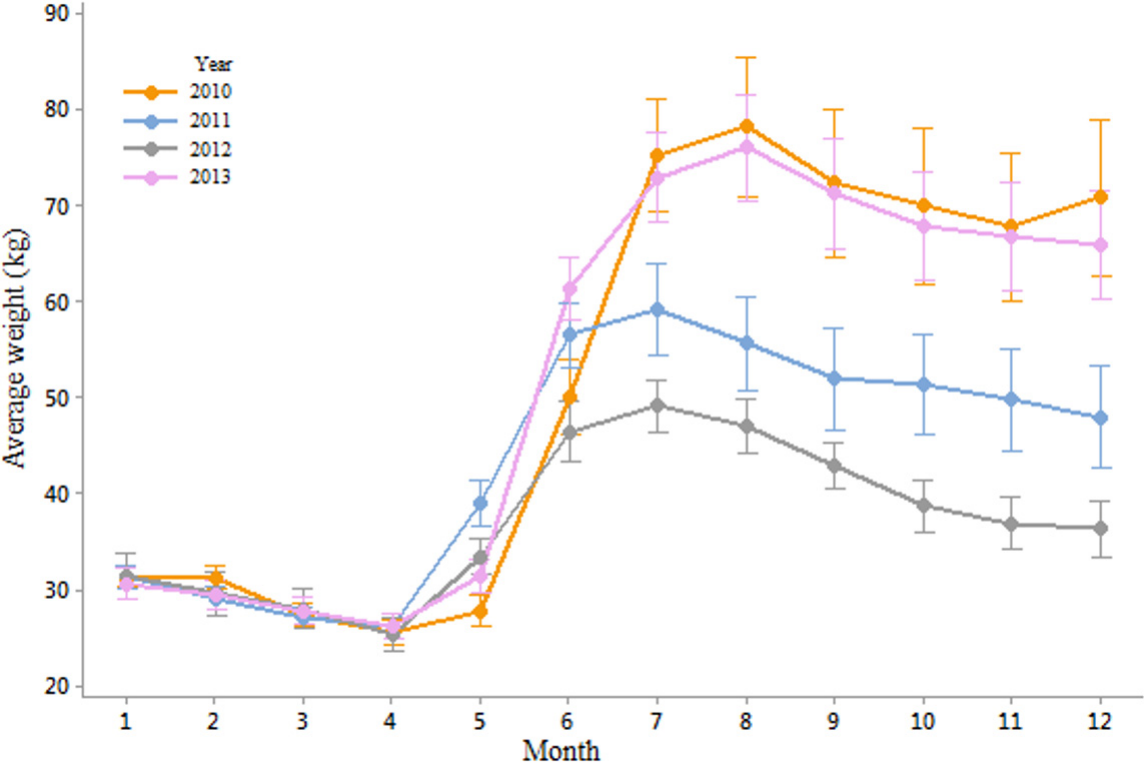
Average monthly hive weights, in kg, for the years 2010, 2011, 2012 and 2013.

**Fig 5:**
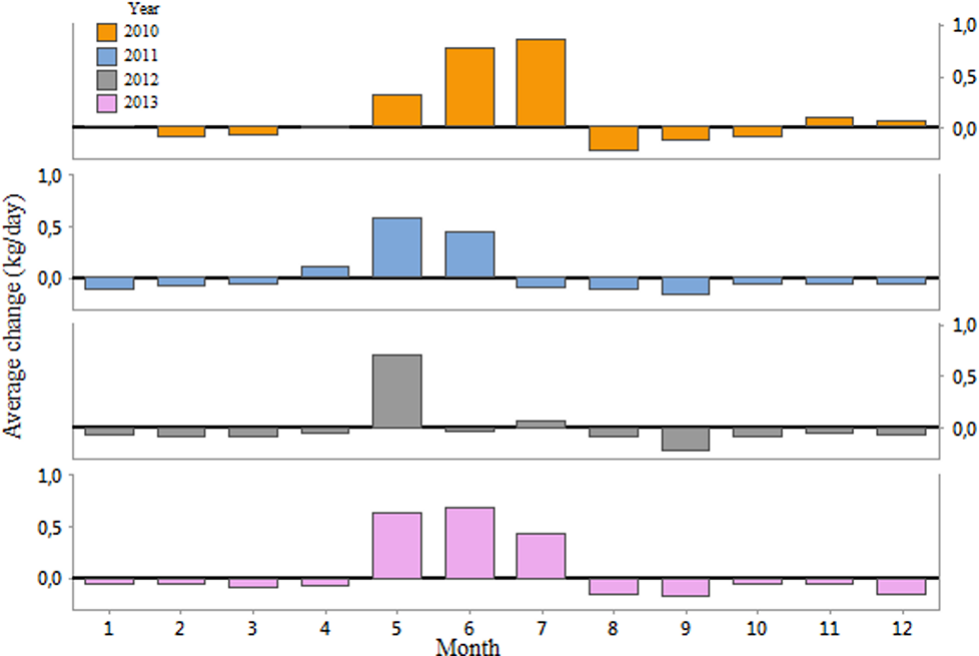
Average monthly change in weight, in kg/day, for the years 2010, 2011, 2012 and 2013.

The monthly average weights showed that the year 2012 was marked by normal average weights in winter and spring but which became lower than in the other years from June onwards (Fig 4). The changes in weight of the hives, from one day to the next, provided us with information regarding the food collection rate or foraging potential (available forage) of bees in productive months of the year, or the consumption rate of colonies during the non-productive months of the year. During the year 2012, colonies were consuming as many resources in winter as those in 2010, 2011, and 2013, and gaining as much weight in early spring. However, this was followed by an average weight loss in June and a shorter productive summer period overall (Fig 5).

### Environmental variables to explain observed colony productivity

The low annual productivity of colonies in the year 2012 appeared to be due to lack of increases in weight in the month of June (-0.03 kg/day compared to 0.77 kg/day, 0.45 kg/day, and 0.69 kg/day in 2010, 2011 and 2013 respectively). In general, the month of June seemed especially important for colony development, considering that in 2011, the colonies lost weight in July (-0.10 kg/day) and August (-0.12 kg/day), but this still resulted in a greater average colony weight than that of 2012 (Fig 4).

**Fig 6:**
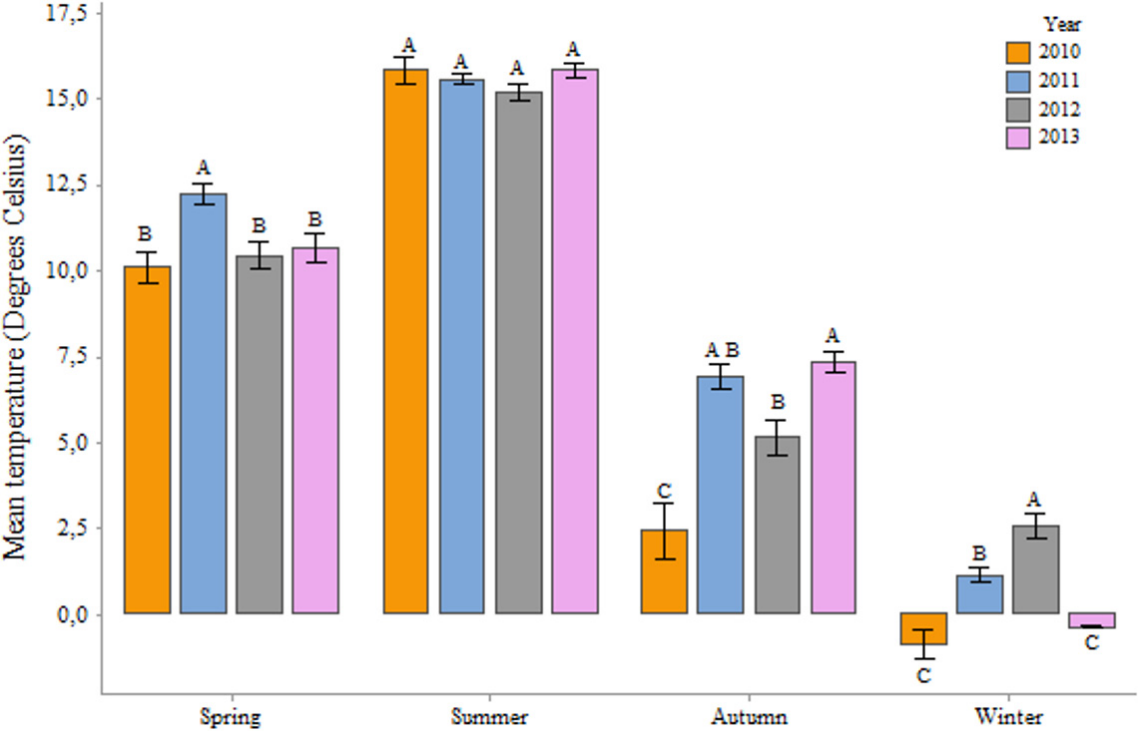
Mean seasonal temperatures in degree Celsius for the whole of Denmark in the years 2010, 2011, 2012 and 2013. Means that do not share a letter are statistically significant. Source: dmi.dk.

**Fig 7:**
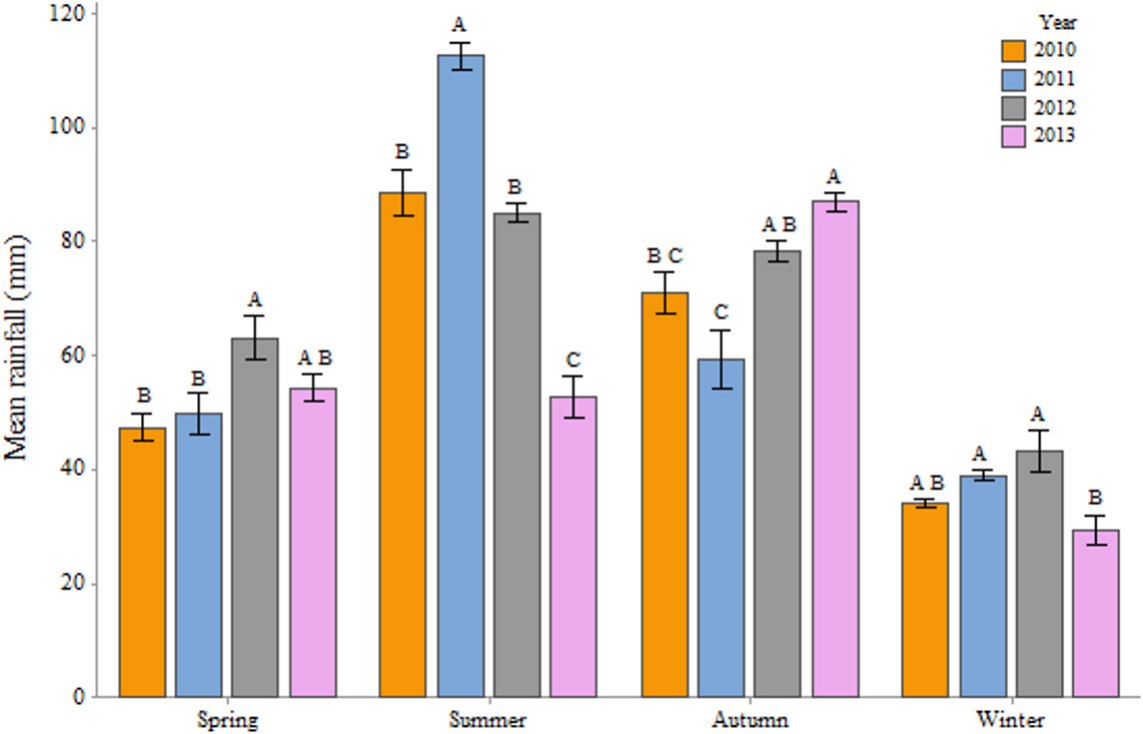
Mean seasonal rainfall in millimeters for the whole of Denmark in the years 2010, 2011, 2012 and 2013. Means that do not share a letter are statistically significant. Source: dmi.dk.

When comparing the environmental variables in the study years, we found significant differences in temperatures between the years (Fig 6) in autumn (One-Way ANOVA; F = 19.55, P< 0.001), spring (One-Way ANOVA; F = 4.41, P = 0.005) and winter (One-Way ANOVA; F = 31.47, P< 0.001) but not in summer (One-Way ANOVA; F = 1.43, P = 0.235). With regards to rainfall (Fig 7), we found significant differences in autumn (One-Way ANOVA; F = 15.64, P< 0.001), spring (One-Way ANOVA; F = 4.72, P = 0.003), summer (One-Way ANOVA; F = 68.19, P< 0.001) and winter (One-Way ANOVA; F = 6.30, P< 0.001). However, monthly averages in temperature and rainfall between months of different years showed even greater variability. June 2012 was punctuated by lower temperature and higher rainfall (12.7 C°; 98 mm) than in 2010 (13.9 C°; 52 mm), 2011 (15.1 C°; 75 mm) and 2013 (14 C°; 68 mm). A similar pattern could be observed in July for the years 2011 and 2012. Negative correlations between rainfall and weight change in June (R = -0.65; P< 0.001) and to a lesser extent July (R = -0.36; P = 0.002) and positive correlations between temperature and weight change in June (R = 0.39; P = 0.001) and July (R = 0.57; P< 0.001) seem to corroborate the results.

### Effects of the immediate landscape on colony productivity

**Fig 8:**
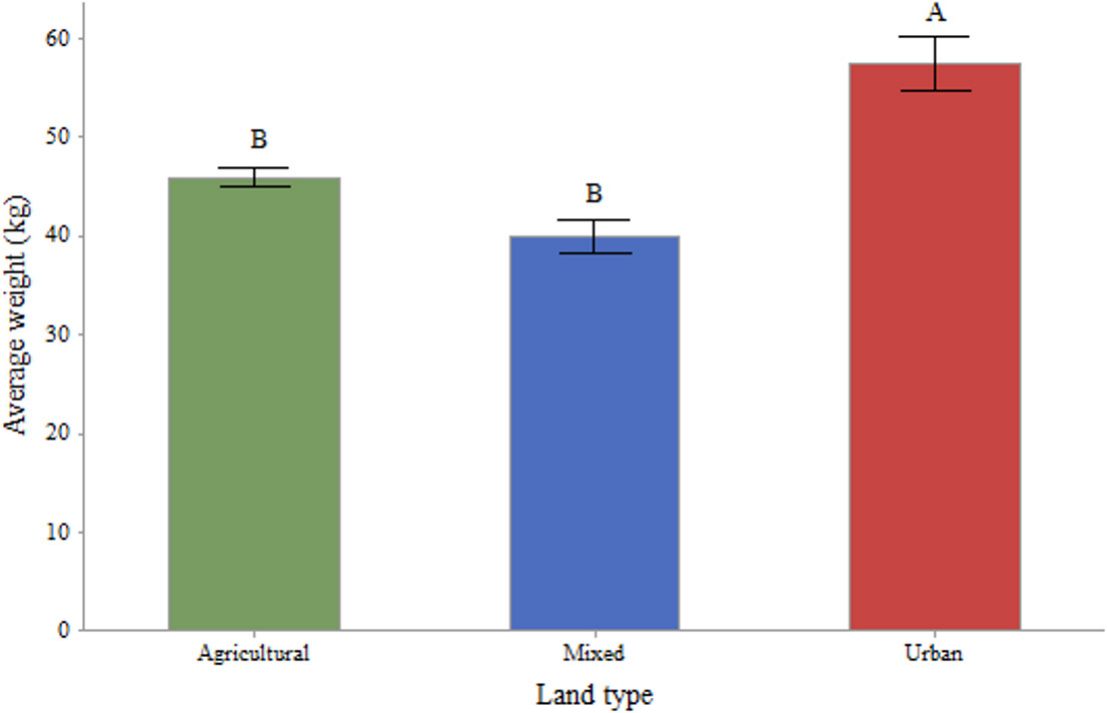
Average hive weights, in kg, by landscape type for a 1km radius around the hives. Agricultural (n = 49; mean = 45.94): More than 50% of the surrounding 1km landscape composed of agricultural areas; Urban (n = 12; mean = 57.50): More than 50% of the surrounding 1km landscape composed of urban areas; Mixed (n = 10; mean = 39.90): A combination of landscape types surrounding the apiary with no single habitat type representing more than 50% of the total landscape. Means that do not share a letter are statistically significant.

**Fig 9:**
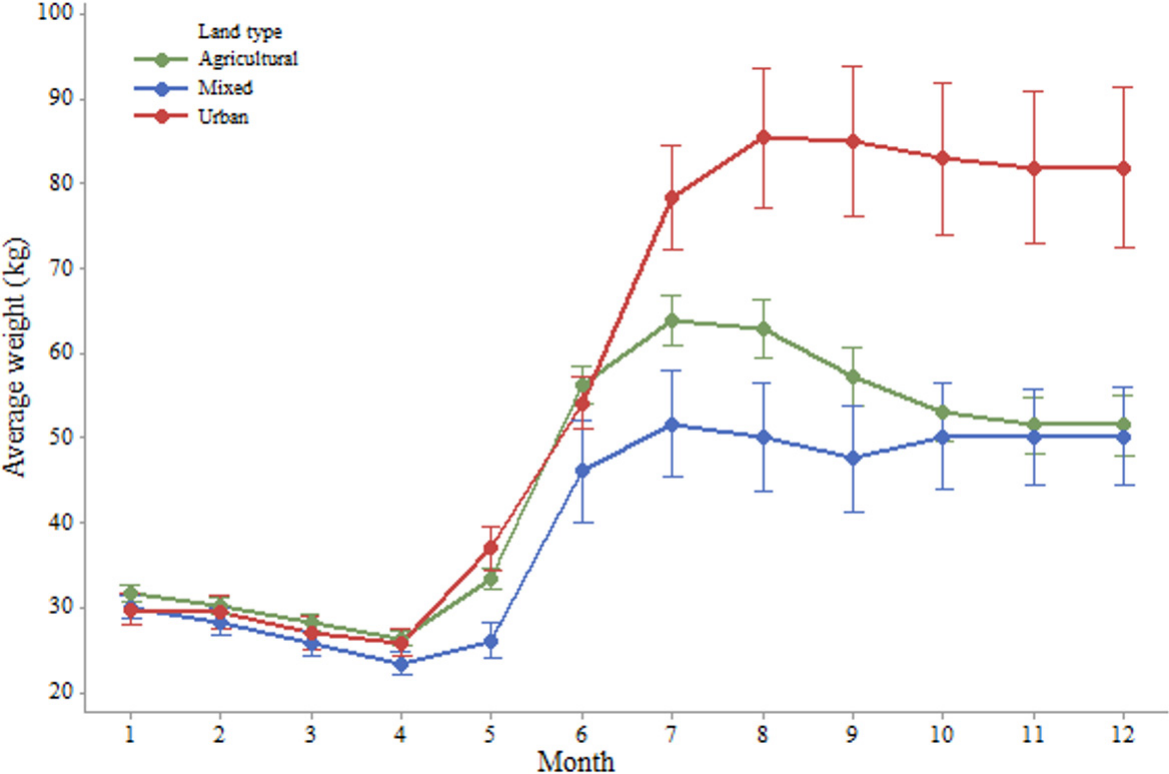
Average monthly hive weights, in kg, by landscape type for a 1km radius circle around the colonies.

**Fig 10:**
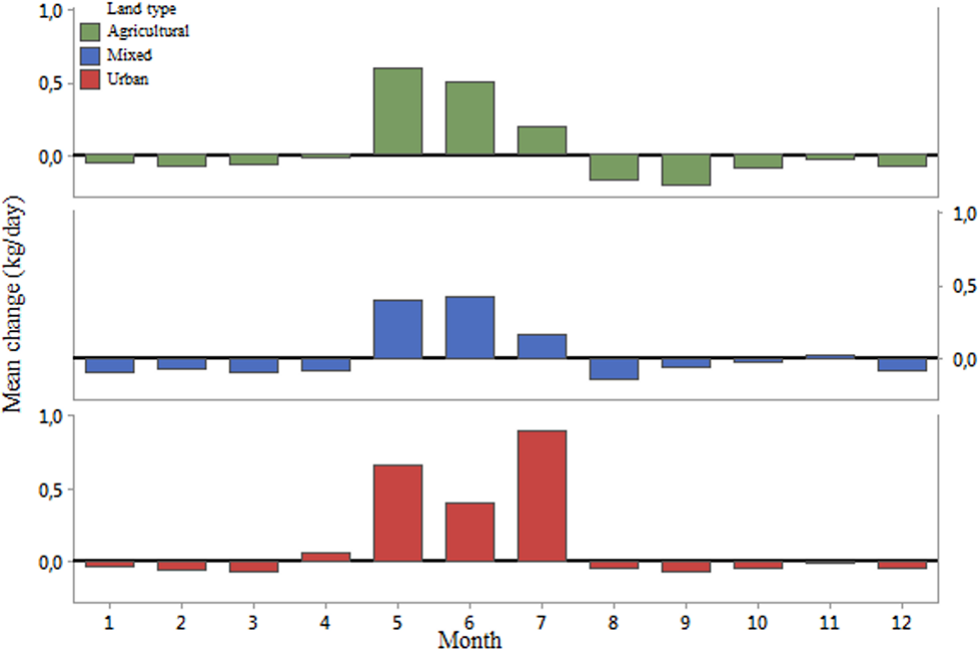
Average monthly change in weight, in kg/day, by landscape type for a 1km radius circle around the hives.

Coupled with apiary locations, and landscape types at the national level provided by the CORINE 2006 data, the data can provide some insight into the effect of the immediate landscape on colony productivity. For a 3km radius (F = 5.213; P = 0.023) and a 1km radius (F = 9.104; P = 0.003) around each apiary, we found significant effects of the proportion of artificial surfaces on the weight of the colonies, but no significant effect of the proportion of agricultural areas at 3km (F = 1.748; P = 0.186) or 1km (F = 3.689; P = 0.055). When we considered the landscape categories as described earlier, the location of each hive significantly impacted the weight of the colony based on the monitoring data at both the 3km (F = 3.11; P = 0.041) and 1km (F = 3.531; P = 0.030) radii. At the 3km radius, hives in urban environments tended to weigh more than their counterparts in mixed (Pairwise Comparison, T = 2.39; P = 0.017) or predominantly agricultural landscapes (Pairwise Comparison, T = 5.60; P = 0.028) (Fig 8). At the 1km radius, hives in urban environment weighed more than those in agricultural landscapes (T = 2.655; P = 0.008). However, at this radius we lost the significant difference with mixed landscapes (T = 1.629; P = 0.104). These differences were also present when comparing the productive months of the year, or the consumption rate of colonies during the non-productive months of the year, with the immediate land types of the hives (Figs 9 and 10). However, significant differences at this level only appeared from the month of July onwards, and through to December.

## Discussion

The distribution and installation of automated electronic scales as a way to monitor the productivity of honey bee colonies has the potential to increase the value of new research and models. In this study, we showed that using the weight of hives, we were able to describe annual patterns of colony productivity from January 2010 to December 2013, and to pinpoint landscape elements of importance to mean colony productivity. In the year 2012, the productivity was significantly lower than in the other years. This difference could be partly explained by the environmental variables; the flight activity of bees is known to be positively correlated with temperature [31], while the sudden onset of cold weather is known to have a negative influence on the development of colonies, resulting in a decline in brood production, even a short duration of bad weather has significant impacts on the honey bee colony [32]. While local weather undeniably directly influences the productivity of a colony, some indirect effects of the climate are just as important. A delayed spring will tend to compress the flowering of fruit trees and oilseed rape, making it difficult for bees to exploit all available resources. In addition, the length of the daily light period (photoperiodism) affects floral induction, with longer days promoting flowering [33]. Therefore, warm, rainy winter or unusually cold spring could affect flowering of the crops which in turn would reduce the nectar flow inside the colonies. Coupled with details on the quantities of honey harvested, and more environmental data, we have the potential to predict future patterns of honey flow and colony growth. The presented results on the monthly losses or gains are valuable in estimating and predicting seasonal variation of the food collection rate of the colonies, which can be used to better understand colony growth and development [34]. The weight gains achieved during May-August represent the bulk of the resources exploited when harvesting honey [35]. The weight fluctuations during this time reflect honey production and to a lesser extent pollen wax and additional bees. We were able to show that this production period can be highly variable in amplitude and dependent on a combination of factors.

With regards to the effect of immediate landscape on colony productivity, we found that bees in urban areas performed better (biomass gain) than their counterparts in agricultural and mixed habitats. By focusing the analysis on the average monthly changes throughout the year, we could see that while the colonies in agricultural, mixed and urban landscapes appeared to perform in a similar manner in winter, their performance differed much more in the productive summer months (Fig 8). The colonies in the agricultural landscapes benefited from crop flowerings during May and June, whereas those in the urban landscapes were able to collect pollen and nectar during a longer period, benefiting potentially from nearby fields but also gardens and parks. In contrast, the colonies in the mixed environments were not able to benefit from significant agricultural or urban areas and might have been left with the resources of the diminishing natural landscape [35]. These findings are in line with previous research as to the potential benefits of good urban planning for bee abundance and diversity [20, 22, 36]. This effect has also been reported in other cities such as Paris [37] or Chicago where the variety of flora in the city is thought to account for the healthier and more productive qualities of the bees [22]. It is worth noting that this greater productivity in an urban landscape might not necessarily be generalised to any urban environment, and should depend on the right conditions, such as flowering botanical gardens, parks, and private backyards filled with flowering trees, bushes and plants [38]. Generally bees are known to be sensitive to agricultural intensification [39], as is becoming more common in Europe and would therefore benefit from their inclusion in urban areas. The value of a seasonal breakdown of the data is clear in its usefulness. Summer has been shown to be the most challenging season for honey bees [13]. Here we show that landscape affects productivity more in certain periods of the year than others. Colonies in all environments performed similarly during the non-productive winter months and the early spring. At the height of the summer though, we were able to demonstrate that the colonies in urban-dominated landscapes are able to forage for longer, whereas in the predominantly agricultural areas, honey bees are restricted to the flowering periods of nearby crops. This result is in line with research from the ECOBEE platform monitoring scheme [14] which identified the period from late May to early July, post oilseed rape flowering period, as a food shortage period for honey bees in intensive agrosytems. As well as the mere presence of flowers during the summer proving to be an advantage for bees foraging in urban areas, the polyfloral nature of this landscape compared to that of the agricultural one (dominated by oilseed rape in Denmark) could also have had an effect on the resulting weights of the colonies [11]. While maize is not a dominating crop in Denmark, its production has increased between 1999 and 2008 and it is becoming common in some of its more southern parts [40]. Höcherl et al [41] showed that bees under dietary stress, consuming a pure maize pollen diet or artificial pollen diets, reduced the amount of brood that they reared, and cannibalized brood. To the extent that maize production occurs in the landscape, it may have contributed to the lower weights observed in the agricultural landscape.

Some of the variation revealed by the data is that of the poor performance of the colony in mixed landscapes. As defined, a mixed landscape is one in which no single habitat type represents more than 50% of the total 1km radius. This class was created because 61 percent of Denmark’s total surface is agricultural land. Based on the location of the apiaries in Denmark; over 68 percent of the 1 km radii are made up of agricultural land, just over 18.5 percent are urban areas; leaving forest and semi-natural habitats with 9.4 percent (water bodies contributed 3.4 percent but are of little consequence to honey bee foraging). As a result, in none of the examined locations are natural habitats even remotely dominant. These locations where neither the agricultural nor the urban landscape types dominate recorded the lowest productions, though only significantly lower than in the urban sites. We do not have any single explanation for this but we could observe that the spring time weight gains of the colonies in mixed landscapes was lower compared to the other two landscape types and they did not benefit from a mid-summer peak as observed in the urban colonies (Fig 10). Hypothetically, a mixed landscape type could have been lacking in flowering crops, dominated more by grain, or grasses, grown in animal production farmlands, and where urbanisation consists mainly of farms or unkempt out-buildings where little attention is placed towards flowering plants. Exploring the performance of colonies located in natural and semi-natural habitats would have been an interesting addition to our research.

While we have shown the value of colony scale monitoring in scientific research, some improvements could still be made to achieve greater reliability of the data. The scales themselves although largely reliable do occasionally breakdown, lose connection to their network, or simply run out of battery. This creates gaps in the data. The environmental variables recorded by the scales (ambient temperature, humidity, rainfall) did not work as reliably as the scales themselves, and their use in analysis remains limited. The reporting of beekeepers’ management practices could simplify automated hivescale data analysis by providing information such as the timing and quantity of honey harvests, the addition of supers, the timing, type and amount of sugar fodder, details of re-queening, pest and pathogens treatments and colony loss. We have been able show that honeybees in urban areas are heavier and more productive than those in agricultural or mixed landscapes; however, we are unable to more than speculate as to the effect of weight on parasite loads, winter survival, or general health of honey bee colonies.

Much of the potential of the value of automated scales still has to be realized. Many European and North American sites have already been fitted with them, and with increased international collaboration, the scales could become an invaluable tool in deepening our understanding of the biology of honey bees and the problems that they face. For example, Wray et al [42] found that colonies that were more active foragers and colonies with greater defensive responses at the start of the summer went on to gain more weight. This means that based on scale data of one summer season, colonies could be selected for specific behavioural traits based on their weight. Finally, diseases of the honeybee monitoring programs are already in place in many countries. National pollen surveys and levels of pesticides in crops are also more frequently established [25]. Coupling these data with colony weights could perhaps help us determine the thresholds and signs of an imminent colony collapse, or the effects of different management techniques on a national scale.

## Supporting Information

S1 Table Monthly regional and national temperatures. A two-sample t-test between each region and the national averages revealed no significant differences in mean temperatures. Source: DMI.dk.
(TIF)

S2 Table Monthly regional and national rainfall. A two-sample t-test between each region and the national averages revealed no significant differences in sum rainfall. Source: DMI.dk.
(TIF)

S3 Table Colony I.D. present for each year of observations, categorized by landscape types at a radius of 1km around the apiary. (TIF)

S4 Table Colony I.D. present for each year of observations, categorized by landscape types at a radius of 3km around the apiary. (TIF)

S5 Table Proportions of land-type surfaces for a 1km radius for each colony I.D. in the study. (TIF)

S6 Table Proportions of land-type surfaces for a 3km radius for each colony I.D. in the study. (TIF)
