# Supplementary figures and images for: Weight Watching and the Effect of Landscape on Honeybee Colony Productivity: Investigating the Value of Colony Weight Monitoring for the Beekeeping Industry
Source: PLoS One. 2015 Jul 6;10(7):e0132473. doi: 10.1371/journal.pone.0132473 (PMC4493132; doi:10.1371/journal.pone.0132473)

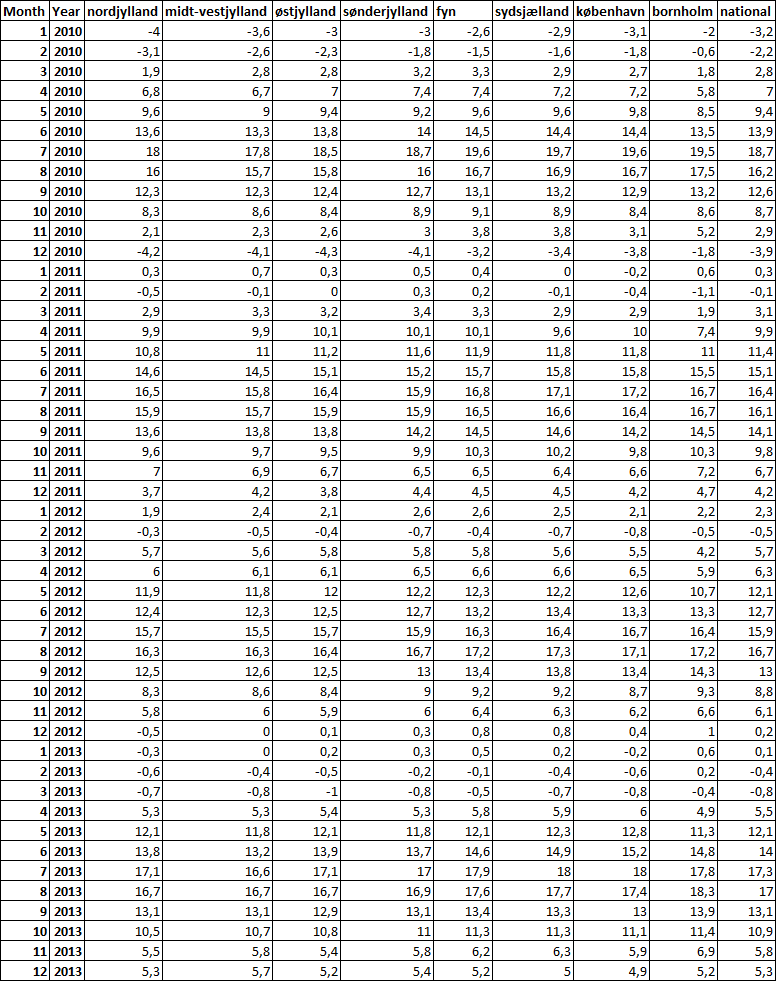

Supplement: S1 Table — A two-sample t-test between each region and the national averages revealed no significant differences in mean temperatures. Source: DMI.dk. (TIF) [file pone.0132473.s001.tif]

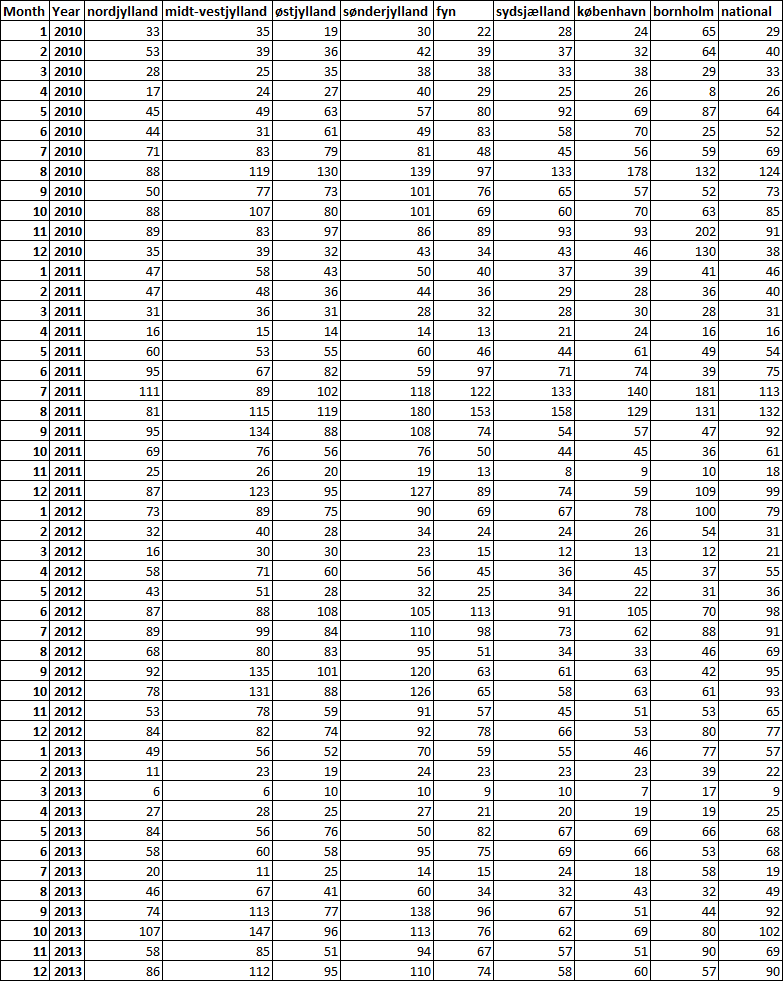

Supplement: S2 Table — A two-sample t-test between each region and the national averages revealed no significant differences in sum rainfall. Source: DMI.dk. (TIF) [file pone.0132473.s002.tif]

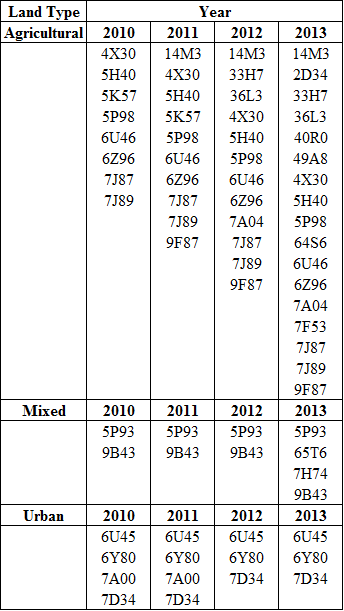

Supplement: S3 Table — (TIF) [file pone.0132473.s003.tif]

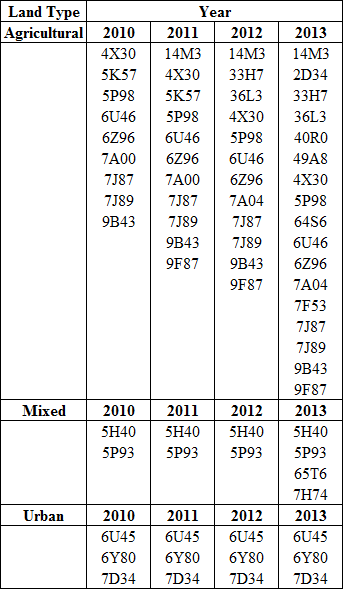

Supplement: S4 Table — (TIF) [file pone.0132473.s004.tif]

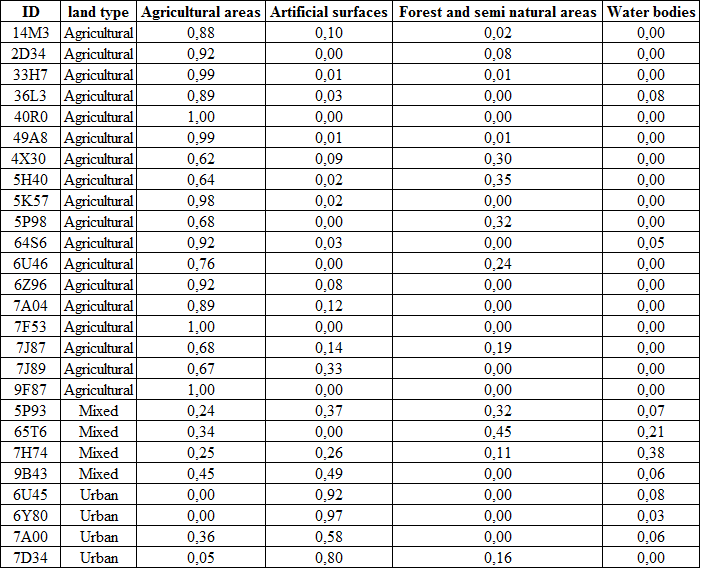

Supplement: S5 Table — (TIF) [file pone.0132473.s005.tif]

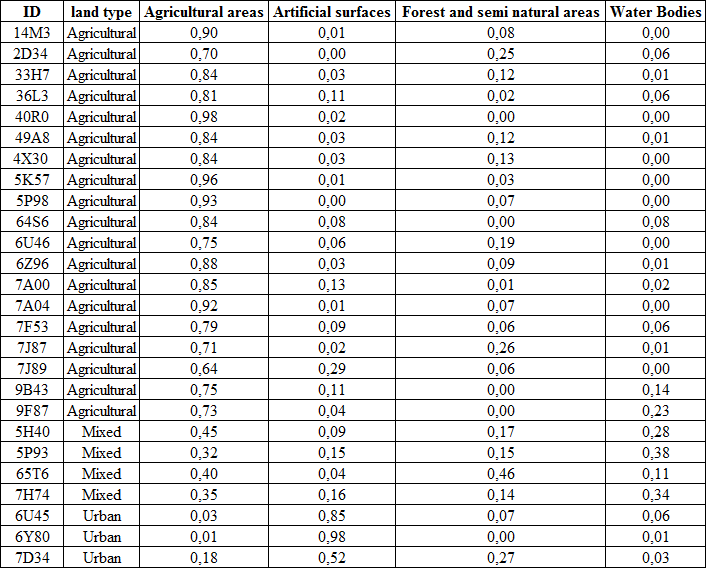

Supplement: S6 Table — (TIF) [file pone.0132473.s006.tif]
